# Effect of the second and third COVID-19 pandemic waves on routine outpatient malaria indicators and case management practices in Uganda: an interrupted time series analysis

**DOI:** 10.1186/s12936-024-05153-0

**Published:** 2024-10-29

**Authors:** Pius Mukisa, Freddy Eric Kitutu, Arthur Mpimbaza, Jaffer Okiring, Joan N. Kalyango, Joaniter I. Nankabirwa

**Affiliations:** 1 Clinical Epidemiology Unit, Makerere University College of Health Sciences, Kampala, Uganda; 2 Child Health and Development Centre, Makerere University, College of Health Sciences, Kampala, Uganda; 3 Department of Pharmacy, Makerere University School of Health Sciences, Kampala, Uganda; 4 Department of Women’s and Children’s Health, International Maternal and Child Health (IMCH), Uppsala University, 751 85 Uppsala, Sweden; 5 Infectious Diseases Research Collaboration, Kampala, Uganda

**Keywords:** Malaria, COVID-19 pandemic, COVID19-wave, Malaria, Outpatient Department, Routine outpatient malaria indicators, Interrupted time series analysis, Malaria case management, Uganda

## Abstract

**Background:**

Reports on the impact of COVID-19 pandemic on the quality of malaria care and burden in sub Saharan Africa have provided a mixed picture to date. The impact of the 2nd (Delta) and 3rd (Omicron) COVID-19 waves on outpatient malaria indicators and case management practices was assessed at three public health facilities with varying malaria transmission intensities in Uganda.

**Methods:**

Individual level data from all patients presenting to the out-patient departments (OPD) of the three facilities (Kasambya, Walukuba and Lumino) between January 2019 and February 2022 were included in the analysis. Outcomes of interest included total number of outpatient (OPD) visits, proportion of patients suspected to have malaria, proportion of suspected malaria cases tested with a malaria diagnostic test, test positivity rates (TPR) and proportion of malaria cases prescribed artemether-lumefantrine (AL). Using the pre-COVID-19 trends between January 2019 and February 2020, interrupted time series analysis was used to predict the expected trends for these study outcomes during the 2nd wave (May 2021–August 2021) and 3rd wave (November 2021–February 2022). The observed trends of the study outcomes were compared with the expected trends.

**Results:**

There were no significant differences between the observed versus expected overall outpatient visits in the 2nd wave, however, a significant decline in OPD attendance was observed during the 3rd wave (15,101 vs 31,154; incidence rate ratio (IRR) = 0.48 [0.41–0.56]). No significant differences in the overall observed versus expected proportions of suspected malaria cases and test positivity rates in both COVID waves. However, a significant decrease in the overall proportion of suspected malaria cases tested with a malaria diagnostic test was observed during the 3rd wave (99.86% vs 99.99%; relative percent ratio [RPR] = 0.99 [0.99–0.99]). Finally, a significant decline in the overall proportion of malaria cases prescribed AL was observed during the 2nd wave (94.99% vs 99.85%; RPR = 0.95 [0.92–0.98]) but not the 3rd wave.

**Conclusion:**

Significant declines in OPD attendance and suspected malaria cases tested with malaria diagnostic test were observed during the 3rd COVID-19 wave, while AL prescription significantly reduced during the 2nd COVID-19 wave. These findings add to the body of knowledge highlighting the adverse impact of COVID-19 pandemic on the malaria which could explain the increase in the malaria burden observed during this period.

## Background

Since 2000, substantial reductions in malaria disease burden have been achieved at global level and in sub-Saharan Africa. However, a rise in malaria case incidence was observed from 2020, part of which was attributable to the COVID-19 pandemic and its disruptions to malaria control interventions [1]. COVID-19 is an infectious disease caused by the SARS-CoV-2 virus [2]. The disease emerged in Wuhan, China in December 2019 and was declared a Public Health Emergency of International concern on January 30th 2020 and a pandemic on March 11th 2020 [3]. This respiratory illness manifests with mild to severe symptoms including fever, cough, difficulty in breathing with severe cases progressing to pneumonia and organ failure [4].

The World Health Organization (WHO) Africa region accounts for majority of the malaria cases reported worldwide [1]. In Uganda, malaria is the leading diagnosis at outpatient departments (OPD) accounting for 31.1% of all OPD visits and the commonest reason for inpatient department (IPD) admissions accounting for ~ 25% of all IPD admissions [5]. In addition, malaria is the 2nd leading cause of death after neonatal conditions, accounting for 7.4% of all inpatient death in the country [5]. Uganda is a malaria endemic country with 95% of the population at risk of infection. Malaria transmission in Uganda varies geographically, from less than 1% malaria prevalence in southwest Uganda to greater than 20% in Busoga subregion, northwestern Uganda, and northeast Uganda [6].

The country has made tremendous progress by reducing the malaria burden with parasite prevalence declining from 42% (based on microscopy in under five children) in 2009 to 9.1% in 2018 [7]. However, an increase in the number of cases was observed in areas previously reporting marked declines in burden starting in 2020 [8, 9]. In 2022, Uganda experienced a rebound epidemic leading with some areas reporting more than a 30% increase in the total number of malaria confirmed cases [8]. This period corresponds to the time the country was having the COVID-19 epidemic, however, the contribution of the epidemic to this increase in burden has not well documented.

Indeed, from the on-set of the COVID-19 pandemic, there were concerns that the documented success in malaria control in Africa may be significantly reversed by the pandemic and modelling studies predicted that malaria cases would double during the pandemic [10]. The impact of COVID-19 on malaria burden could be through a number of mechanisms including disruptions in health-seeking behaviours, reallocation of resources, misdiagnosis due to overlap of symptoms, and interruptions in malaria preventive services [11, 12].

In Uganda, the first COVID-19 case was registered on 21st March 2020 [13], and three waves were observed through the course of the pandemic. The 1st COVID-19 was between August 2020 to January 2021, 2nd wave between May 2021 and August 2021 and the 3rd wave between November 2021 and February 2022 [14, 15]. A study by Namuganga et al*.* showed no impact of the 1st COVID-19 wave on malaria burden [16], however, this study was done when the number of reported COVID-19 cases in Uganda were low and less severe in presentation. The 2nd COVID-19 wave (Delta) in Uganda had more severe cases [17] and the 3rd COVID-19 wave (Omicron wave) had more infectious cases. Despite these differences in presentation to the first wave, the impact of the 2nd and 3rd wave on malaria burden and case management have not been evaluated. In this study, the effect of the 2nd and 3rd COVID-19 waves on routine outpatient malaria indicators and case management practices was assessed at three public health facilities located in varying malaria transmission settings in Uganda.

## Methods

### Study design and setting

This was a time trend analysis of malaria burden indicators and case management practices using data of patients attending out-patient departments of three public health facilities in Uganda. The health facilities included two level III health centres (Kasambya and Lumino) and one level IV health centre (Walukuba). All facilities are part of 77 malaria reference centres (MRCs) in Uganda, where enhanced malaria surveillance activities are conducted as part of routine surveillance. The three facilities are supported by the Uganda Malaria Surveillance Project (UMSP) as part of MRC activities to capture accurate, reliable and complete individual patient level data, using the standardized health management information system (HMIS) registers (HMIS 002 outpatient register). Staff capacity building is provided through training, onsite mentorship, support supervision and regular data quality assessments.

The facilities attend to between 1000 and 3000 outpatients monthly. The main malaria control interventions in the districts have been limited to the use of long-lasting insecticidal nets (LLINs) and to date there have been four mass net distribution campaigns (2013, 2017, 2020 and 2023). Kasambya HC III is located in Mubende district, in the Central Region of Uganda. Mubende is one of the largest districts in the country with agriculture being the main economic activity of the population in the district. The entomological inoculation rate (EIR) of Mubende district is estimated at 4 infective bites per person per year [18] and malaria parasite prevalence in children under 5 years of age was estimated at of 9% in the last malaria indicator survey [6], and it is considered to be a moderate malaria transmission area.

Lumino HC III is located in Busia district in eastern Uganda. Busia is a rural district, with high malaria transmission and the EIR was estimated at 108.2 infective bites per person per year in 2020 [19]. Walukuba HC IV is located in Jinja district in east central Uganda. The district is semi-urban with varying levels of malaria transmission intensities. The malaria parasite prevalence of the district was estimates at 21% in under 5 years in the 2018/19 MIS [6]. The EIR of the area is 6 infective bites per person per year [18].

### Study population, sampling and sample size

All records of patients presenting to the outpatients department of the participating facilities between January 1st 2019 to February 28th 2022 were utilized in the study. The routinely collected data in registers including patient demographics, village of residence, history of fever, whether a malaria diagnostic test was performed, type of malaria test done (malaria rapid diagnostic test (RDTs) vs microscopy), results of laboratory tests, diagnoses given, and treatments prescribed was extracted from the routine HMIS registers. The outcome variables included total OPD visits, suspected malaria cases, TPR, proportions of suspected malaria cases for whom a malaria laboratory test was recommended, and proportion of confirmed malaria cases prescribed AL.

The main exposure variable was the time period in which a patient presented to the OPD (before the COVID-19 pandemic, or during the 2nd or 3rd COVID-19 wave). The potential confounders controlled for in this study included rainfall distribution and temperature. The data on average monthly temperature and rainfall was extracted from remote sensing sources. Rainfall data was extracted from climate hazards group infrared precipitation with station data (CHRIPS) database which data is recorded in millimeters. Temperature data was extracted from the moderate resolution imaging spectro-radiometer (MODIS).

### Data analysis

Single group Newey approach interrupted time series analysis (ITSA) with two interruptions [20] was conducted using STATA 14. Monthly time points were considered, utilizing monthly aggregated data collected from January 2019 to February 2022 for each outcome. The two interruption time points included; (1) the month of onset of the 2nd COVID-19 wave (May 2021) and (2) the time of onset of the 3rd COVID-19 wave (November 2021) in Uganda. The 1st interruption (2nd COVID-19 wave) begun in May 2021 and continued until August 2021 therefore, it had 4 time points in its post interruption duration. There was a wash out period of 2 months (September 2021 to October 2021) before onset of the 2nd interruption (3rd COVID-19 wave). The 2nd interruption had 2 time points in its post interruption period (December 2021 to February 2022).

The Single group newey approach ITSA with two interruptions model output is as follows; Y_t_ = β_0_ + β_1_T + β_2_f_1_ + β_3_f_1_T_1_ + β_4_f_2_ + β_5_f_2_T_2_ + Et, where Et = β_6_Dt + β_7_Tet + β_10_Rt, Yt is outcome Y (e.g., total OPD visits, proportion suspected malaria cases, test positivity rate, proportion malaria cases prescribed AL, proportion of suspected malaria cases tested) at month t, β_0_ is the intercept (outcome Y at the beginning of the study), β_1_ is the slope of the outcome before arrival of the 1st interruption (pre-intervention slope), β_2_ is the change in level of the outcome immediately on arrival of the first interruption, β_3_ is the difference between the pre-intervention slope (pre-COVID-19 slope) and the first interruption outcome slope, β_4_ is the change in the level of slope of the outcome on arrival of the second interruption (3rd COVID-19 wave), β_5_ is the difference between the first interruption (2nd COVID-19 wave) and second interruption slopes (3rd COVID-19 wave slope) of the outcome.

T is a linear term denoting the duration since the start of the study. F_1_t is a linear term denoting the time in month since the start of the 2nd COVID-19 wave (models the observed change in trend/slope immediately after onset of the 2nd COVID-19 wave). F_2_t is a linear term denoting the time in month since the start of the 3rd COVID-19 wave (models the observed change in trend/slope immediately after onset of the 3rd COVID-19 wave, f_1,_ and f_2_ are dummy variables depicting the interventions.

Dt is a linear term denoting fixed calendar month effects to model seasonality, Tet is a linear term of monthly temperature data averaged across district level to control for confounding effects of temperature, Rt is a linear term of monthly rainfall data(mm) averaged across district level to control for confounding effects of rainfall. To account for serial autocorrelation between time points, an autoregressive order two (Lag 2) was used since autocorrelation was present at lags < 2.

Negative binomial was used to model the relationship between the count outcome (OPD visits) and the various independent variables (time, confounders and interruptions indicators). In the same way, fractional regression was used to model the proportional outcomes. Monthly expected values (counterfactual values) of all the outcomes hadn’t the interruptions occurred were predicted based on the fixed model (negative binomial and fractional regression) after adjusting for the calendar month effects, rainfall, temperature and setting the post interruption slopes at zero (to model what would happen if the interruptions hadn’t occurred). For count outcomes, monthly expected values were summed up for the durations of the 2nd and 3rd COVID-19 waves and incidence rate ratios calculated comparing the observed versus expected outcome. For proportion outcomes, an average from the monthly expected values was calculated and a relative percent ratio calculated comparing the observed versus expected outcome.

Significant change in level of the outcome meant immediate impact of the disruption. Significant difference between the pre-intervention and post intervention outcome slopes meant impact of the intervention/disruption overtime. Significant differences between the observed and predicted post intervention outcome values also indicated impact on the intervention.

## Results

A total of 180,666 patients were treated at the outpatients department of the three facilities between 1st January 2019 and 28th February 2022. Most were female 118,815 (65.75%) and the median age of the patients was 16 (6–32) years. The average atmospheric temperature across the three study sites was 30.25 (± 5.49)˚C ranging from 30.8˚C to 32.5˚C. The average rainfall distribution at the sites was 140.4 (± 77.21) mm with the lowest 104.81 mm and the highest 169.88 mm. as shown in Table 1.

**Table 1:** Patient demographics and environmental characteristics stratified by site from January 2019 to February 2022

	Lumino	Kasambya	Walukuba	All sites combined
Median Age (IQR)	17 (5–31)	16 (6–32)	15 (7–33)	16 (6–32)
Female (n%)	42,803 (67.81%)	31,189 (64.34%)	44,346 (65.65%)	118,815 (65.75%)
Male (n%)	20,319 (32.19%)	17,287 (35.66%)	23,204 (34.35%)	61,849 (34.23%)
Average temperature in˚C (SD)	31.88 (2.51)	30.83 (2.65)	32.53 (1.96)	30.25 (5.49)
Average rainfall in mm (SD)	169.88 (101.29)	104.81 (52.35)	146.61 (89.24)	140.43 (77.21)

### Impact of the COVID-19 waves on outpatient malaria indicators

#### OPD attendance (overall impact)

**Fig. 1:**
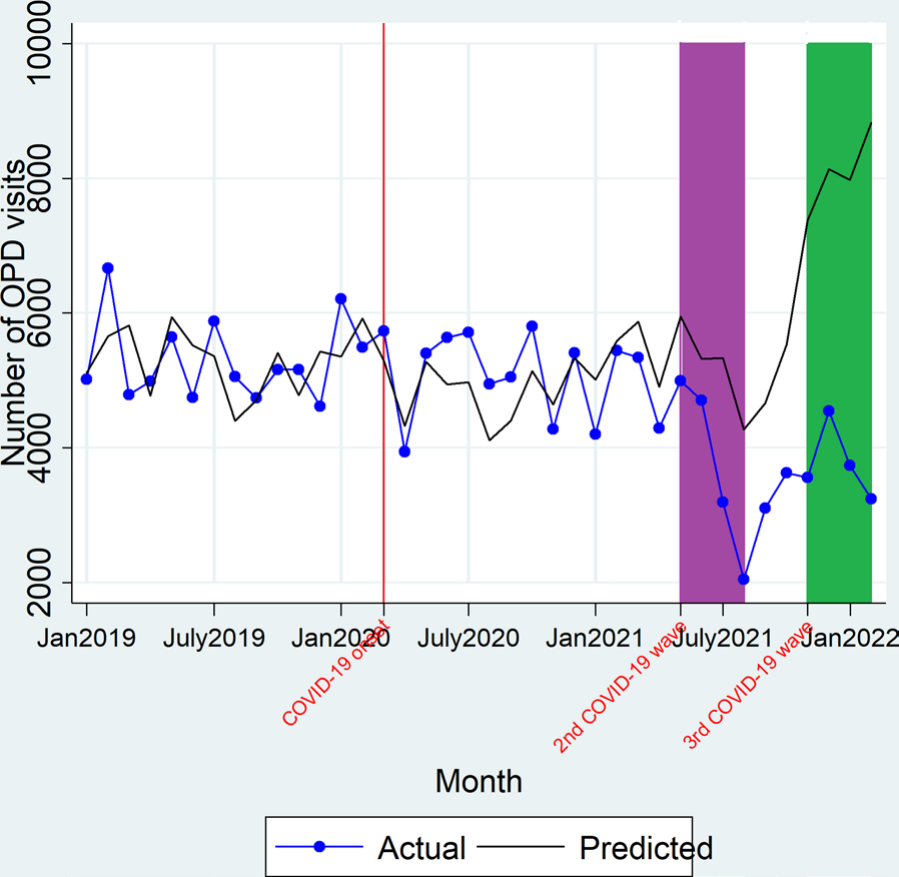
Actual and predicted OPD visits during the study duration. Red line denotes March 2020, the time when the country had its first COVID-19 cases and institution of restrictive measures on transport and lockdowns. Purple block depicts the duration when the country had the 2nd COVID-19 wave (May 2021–August 2021). Green block denotes the duration covered by the 3rd COVID-19 wave (November 2021–February 2022)

There was neither change in level of OPD visits a month immediately on onset of the 2nd COVID-19 wave (β_2_ = − 626.06, P > 0.05) nor change in trend of OPD visits during the 2nd COVID-19 wave duration (β_3_ = − 192.89, P > 0.05). However, immediately on onset of the 3rd COVID-19 wave (during its 1st month), there was a significant increase in the OPD visits (β_2_ = 1532.91, P = 0.03) but overtime there was no significant change in trend of OPD visits during the 3rd COVID-19 wave (β_3_ = − 161.39, P > 0.05) as shown in Fig. 1 and Table 2. Overall (all sites combined), there was no significant difference between the observed versus expected total number of patients seen at the out-patient departments during the 2nd COVID-19 wave (14,950 vs 20,016; IRR = 0.75[0.29–1.20]) however, there was a 52% decline in the number of observed versus expected total number of patients seen at the out-patient departments during the 3rd COVID-19 wave duration (15,101 vs 31,154; IRR = 0.48 [0.41–0.56]) as shown in Table 3.

**Table 2:** Change in level of the outcome and change in trend of the outcome on onset of the two interruptions

	2nd COVID-19 wave onset (May 2021)		3rd COVID-19 wave onset (November 2021)	
	Change in level/β_2_ (P-value)	Change in trend/β_3_ (P-Value)	Change in level/β_2_ (P-value)	Change in trend/β_3_ (P-Value)
OPD attendance	− 626.06 (0.22)	− 192.89 (0.13)	1532.91 (0.03)	− 161.39 (0.44)
Proportion of suspected malaria cases	0.37 (0.86)	− 0.25 (0.55)	− 4.51 (0.09)	2.55 (0.01)
Test positivity rate	− 0.16 (0.00)	0.02 (0.08)	− 0.01 (0.88)	− 0.04 (0.15)
Proportion of suspected malaria cases tested	− 3.84e− 16 (0.51)	7.95e− 18 (0.96)	− 4.11e−16 (0.43)	− 2.77e−16 (0.41)
Proportion of malaria cases treated	3.33e− 14 (0.34)	7.66e− 16 (0.95)	5.31e−14 (0.24)	1.21e−14 (0.60)

**Table 3:** Estimated and observed outcomes (averages for proportion outcomes and totals for the count outcome) during the three interruption durations (all sites combined)

	2nd COVID-19 wave duration (May–August 2021)			3rd COVID-19 wave duration (November 2021–February 2022)		
	Observed	Predicted	Ratio (95% CI)	Observed	Predicted	Ratio (95% CI)
OPD attendance	14,950	20,016	0.75 (0.29–1.20)	15,101	31,154	0.48 (0.41–0.56)
Proportion of suspected malaria cases	36.45%	36.44%	1.00 (0.92–1.08)	35.25%	33.69%	1.05 (0.95–1.13)
Test positivity rate	35.30%	33.89%	1.04 (0.74–1.34)	40.16%	36.28%	1.11 (0.90–1.31)
Proportion of suspected malaria cases tested	99.47%	99.97%	0.99 (0.98–1.00)	99.86%	99.99%	0.99 (0.99–0.99)
Proportion of malaria cases treated	94.99%	99.85%	0.95 (0.92–0.98)	96.96%	99.93%	0.97 (0.94–1.00)

#### OPD attendance (site specific impact)

There was no significant difference between the observed versus predicted OPD visits at a Lumino HCIII for the 2 durations; (1) 2nd COVID-19 wave (5277 vs 4698; IRR = 1.12 [0.86–1.39]) and (2) 3rd COVID-19 wave (5391 vs 5332; IRR = 1.01 [0.96–1.06]) as shown in Table 4. However, there was a 37% decline (7849 vs 13,931; IRR = 0.63 [0.57–0.70]) and 59% decline (3832 vs 9297; IRR = 0.41 [0.37–0.45]) in OPD visits during the 2nd COVID-19 wave duration and 3rd COVID-19 wave duration, respectively at Kasambya HCIII as shown in Table 5. At Walukuba HCIV, there was a 46% decline (4770 vs 8768; IRR = 0.54 [0.15–0.94]) in OPD visits during the 2nd COVID-19 wave and a 67% decline (4950 vs 15,065; IRR = 0.33 [0.23–0.43]) during the 3rd COVID-19 wave as shown in Table 6.

**Table 4:** Estimated and observed outcomes (averages for proportion outcomes and totals for the count outcome) during the three interruption durations (high malaria transmission intensity site)

	2nd COVID-19 wave duration (May–August 2021)			3rd COVID-19 wave duration (November 2021–February 2022)		
	Observed	Predicted	Ratio(95% CI)	Observed	Predicted	Ratio(95% CI)
OPD attendance	5277	4698	1.12 (0.86–1.39)	5391	5332	1.01 (0.96–1.06)
Proportion of suspected malaria cases	78.24%	79.08%	0.99 (0.97–1.01)	82.53%	78.45%	1.05 (0.99–1.01)
Test positivity rate	48.40%	36.71%	1.31 (0.70–1.93)	59.91%	32.29%	1.19 (1.65–2.06)
Proportion of suspected malaria cases tested	99.34%	99.93%	0.99 (0.98–1.01)	100.00%	100.00%	
Proportion of malaria cases treated	90.57%	18.48%	4.92 (-6.85–16.69)	95.02%	0.24%	397.28 (− 796.51 to 7811.09)

**Table 5:** Estimated and observed outcomes (averages for proportion outcomes and totals for the count outcome) during the three interruption durations (moderate malaria transmission intensity site)

	2nd COVID-19 wave duration (May–August 2021)			3rd COVID-19 wave duration (November 2021–February 2022)		
	Observed	Predicted	Ratio (95% CI)	Observed	Predicted	Ratio (95% CI)
OPD attendance	7849	13,931	0.63 (0.57–0.70)	3832	9297	0.41 (0.37–0.45)
Proportion of suspected malaria cases	60.12%	75.87%	0.79 (0.32–1.26)	60.79%	76.28%	0.79 (0.72–0.87)
Test positivity rate	22.19%	34.30%	0.65 (0.41–0.88)	29.21%	40.16%	0.73 (0.63–0.83)
Proportion of suspected malaria cases tested	97.59%	99.72%	0.99 (0.61–1.39)	99.79%	99.97%	0.99 (0.99–0.99)
Proportion of malaria cases treated	90.57%	18.40%	4.92 (− 10.06 to 19.90)	95.57%	0.24%	392.29 (− 5420.98 to 6215.56)

**Table 6:** Estimated and observed outcomes (averages for proportion outcomes and totals for the count outcome) during the three interruption durations (low malaria transmission intensity site)

	2nd COVID-19 wave duration (May–August 2021)			3rd COVID-19 wave duration (November 2021–February 2022)		
	Observed	Predicted	Ratio(95% CI)	Observed	Predicted	Ratio(95% CI)
OPD attendance	4770	8768	0.54 (0.15–0.94)	4950	15,065	0.33 (0.23–0.43
Proportion of suspected malaria cases	41.12%	34.99%	1.18 (0.48–1.87)	30.76%	14.54%	2.12 (2.07–2.16)
Test positivity rate	49.36%	55.02%	0.89 (0.63–1.16)	47.53%	32.92%	1.44 (0.74–2.15)

#### Proportion of suspected malaria cases (overall impact)

**Fig. 2:**
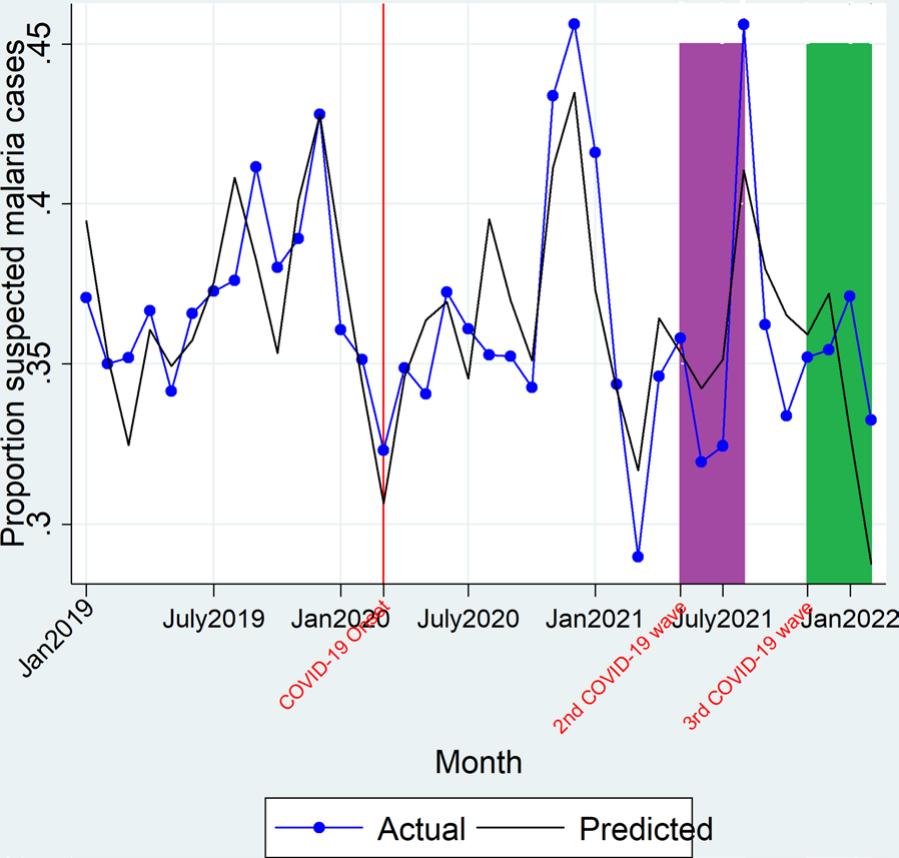
Actual and predicted proportion of suspected malaria cases. Red line denotes March 2020, the time when the country had its first COVID-19 cases and institution of restrictive measures on transport and lockdowns. Yellow block denotes the duration covered by the 1st COVID-19 wave (August 2020–January 2021), Purple block depicts the duration when the country had the 2nd COVID-19 wave (May 2021–August 2021). Green block denotes the duration covered by the 3rd COVID-19 wave (November 2021–February 2022)

On onset of the 2nd COVID-19 wave, there was neither an immediate significant change in the level (β_2_ = 0.37, P > 0.05) of the proportion of suspected malaria cases nor a significant change in trend (β_3_ = − 0.25, P > 0.05) of the proportion of suspected malaria cases during the 2nd COVID-19 wave. There was no significant change in the level (β_2_ = − 4.51, P > 0.05) of the proportion of suspected malaria cases on onset of the 3rd COVID-19 wave however, there was a significant change in trend (β_3_ = 2.55, P = 0.01) with an increment in the proportion of suspected malaria cases during the 3rd COVID-19 wave duration as shown in Fig. 2 and Table 2. Overall, there was no significant differences between the observed versus predicted mean proportion of suspected malaria cases during the 2nd COVID-19 wave duration (36.45% vs 36.44%; RPR = 1.00 [0.92–1.08]). Likewise, there were no significant differences between the observed versus predicted mean proportion of suspected malaria during the 3rd COVID-19 wave (35.25% versus 33.69%; RPR = 1.05 [0.95–1.13]) as shown in Table 3.

#### Proportion of suspected malaria cases (site specific impact)

At Lumino HCIII, there was no significant difference between the observed versus predicted proportion of suspected malaria cases; (1) during the 2nd COVID-19 wave (78.24% versus 79.08%; RPR = 0.99 [0.97–1.01]) and (2) during the 3rd COVID-19 wave (82.53% versus 78.45%; RPR = 1.05 [0.99–1.01]) as shown in Table 4. At Kasambya HCIII, there was a 21% decline in the proportion of suspected malaria cases during the 3rd COVID-19 wave (60.79% versus 76.28%; RPR = 0.79 [0.71–0.87]) however, during the 2nd COVID-19 wave duration, there was no significant difference between the observed versus predicted (60.12% versus 75.87%; RPR = 0.79 [0.32–1.26]), proportion of suspected malaria cases at the moderate malaria transmission site as depicted in Table 5. At Walukuba HCIV, there were no significant differences between the observed versus the predicted proportion of suspected malaria cases tested during the 2nd COVID-19 wave duration (49.36% versus 55.02%; RPR = 0.89 [0.63–1.16]). However, during the 3rd COVID-19 wave duration, the observed suspected malaria cases were significantly higher than expected (30.76% versus 14.54%; RPR = 2.12 [2.07–2.16]).

#### Test positivity rate (overall impact)

**Fig. 3:**
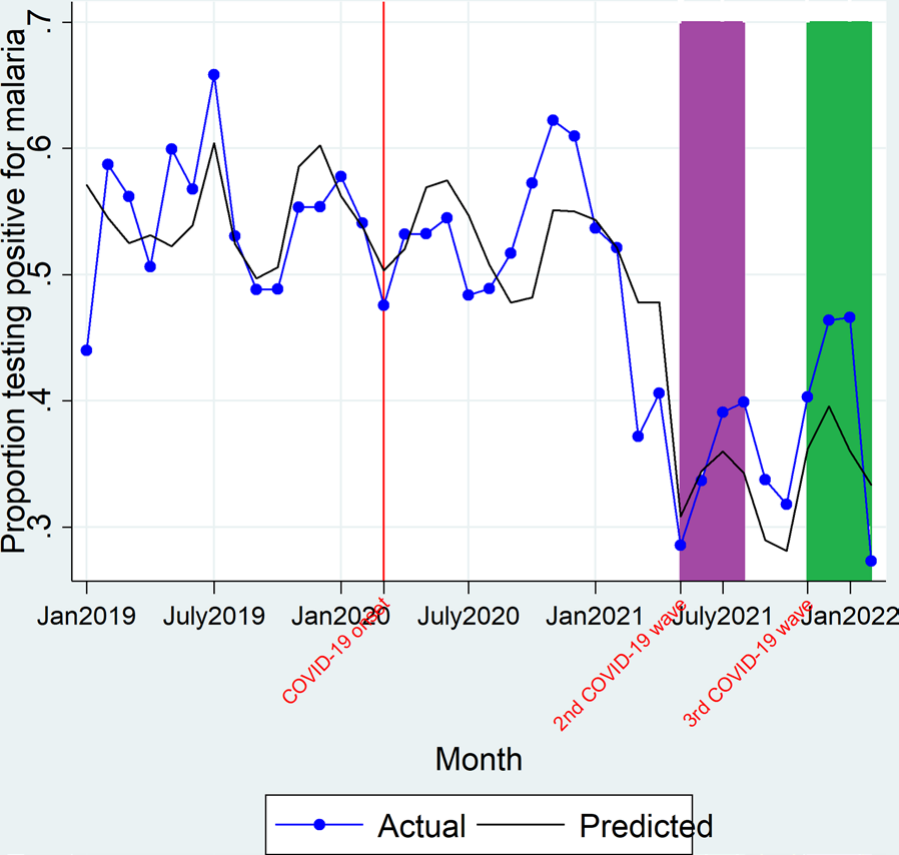
Actual and predicted malaria test positivity rate. Red line denotes March 2020, the time when the country had its first COVID-19 cases and institution of restrictive measures on transport and lockdowns. Purple block depicts the duration when the country had the 2nd COVID-19 wave (May 2021–August 2021). Green block denotes the duration covered by the 3rd COVID-19 wave (November 2021–February 2022)

On onset of the 2nd COVID-19 wave, there was an immediate significant decline (β_2_ = − 0.16, P = 0.00) in the malaria TPR as depicted in Fig. 3 and Table 2. However, there wasn’t a significant change in trend (β_3_ = 0.02, P > 0.05) of the TPR during the entire 2nd COVID-19 wave duration. In the same way, there wasn’t a significant change in level (β_2_ = − 0.01, P > 0.05) of the malaria TPR immediately on onset of the 3rd COVID-19 wave nor was there a significant change in trend (β_3_ = − 0.04, P > 0.05) of the malaria TPR during the 3rd COVID-19 wave duration as shown in Table 2. Overall, there was no significant difference between the observed versus expected malaria TPR (all sites of varying malaria transmission intensities combined) during the 2nd COVID-19 wave (35.30% vs 33.89%; RPR = 1.04 [0.74–1.34]) and during the 3rd COVID-19 wave (40.16% vs 36.28%; RPR = 1.11 [0.90–1.31]) as shown in Table 3.

#### Test positivity rate (site specific impact)

At Lumino HCIII, there was no significant difference between the observed versus the predicted malaria TPR during the 2nd COVID-19 wave duration (48.40% vs 36.71%; RPR = 1.31 [0.70–1.93]). However, during the 3rd COVID-19 wave, the observed TPR was significantly higher (59.91% vs 32.29%; RPR = 1.19 [1.65–2.06]) than expected as shown in Table 4. At Kasambya HCIII, there was a 35% decline and 27% decline in the malaria TPR during the 2nd COVID-19 wave and 3rd COVID-19 wave duration, respectively at this site as shown in Table 5. At Walukuba HCIV, there was no significant difference between the observed versus predicted malaria TPR for the three durations; (1) during the 2nd COVID-19 wave duration (49.36% vs 55.02%; RPR = 0.89 [0.63–1.16]), (2) and during the 3rd COVID-19 wave duration (47.53% vs 32.92%; RPR = 1.44 [0.74–2.15]) as shown in Table 6.

### Impact of the COVID-19 waves on the proportion of suspected malaria cases tested

#### Overall impact

**Fig. 4:**
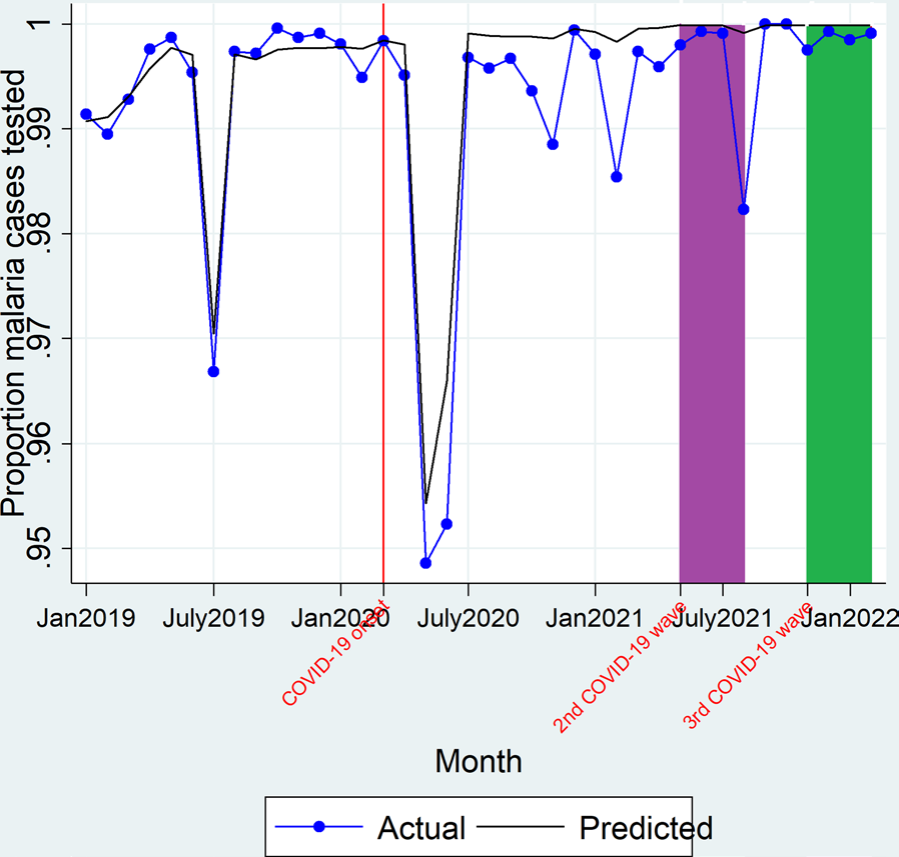
Actual and predicted proportion of suspected malaria cases tested. Red line denotes March 2020, the time when the country had its first COVID-19 cases and institution of restrictive measures on transport and lockdowns. Purple block depicts the duration when the country had the 2nd COVID-19 wave (May 2021–August 2021). Green block denotes the duration covered by the 3rd COVID-19 wave (November 2021–February 2022)

On onset of the 2nd COVID-19 wave, there was neither an immediate change in level (β_2_ = − 3.84e−16, P > 0.05) of the proportion of suspected malaria cases tested nor a significant change in trend (β_3_ = − 7.95e−18, P > 0.05) of the proportion of the suspected malaria cases tested during the 2nd COVID-19 wave duration. There was neither a significant change in the level (β_2_ = − 4.11e−16, P > 0.05) of the proportion of suspected malaria cases tested on onset of the 3rd COVID-19 wave nor a significant change in the trend (β_3_ = − 2.77e−16, P > 0.05) of the proportion of suspected malaria cases tested during the 3rd COVID-19 wave duration as shown in Fig. 4 and Table 2. Overall, there was both a 1% decline in the proportion of suspected malaria cases tested during the 3rd COVID-19 wave duration (99.86% vs 99.99%; RPR = 0.99 [0.99–0.99]). However, there was no significant difference between the observed versus predicted mean proportion of tested malaria during the 2nd COVID-19 wave (99.47% vs 99.97%; RPR = 0.99 [0.98–1.00]) as shown in Table 3.

#### Site specific impact

At Lumino HCIII, there were no significant differences between the observed versus predicted proportion of suspected malaria cases tested during the all the two interruption durations as shown in Table 4. Likewise, there was no significant differences between the observed versus predicted proportion of suspected malaria cases tested at Kasambya HCIII during the 2nd COVID-19 wave, but, during the 3rd COVID-19 wave duration, there was a 1% decline in the proportion of suspected malaria cases tested (99.79% vs 99.97%; RPR = 0.99 [0.99–0.99]) as shown in Table 5.

### Impact of the COVID-19 waves the proportion of malaria cases prescribed artemether lumefantrine (AL)

#### Overall impact

**Fig. 5:**
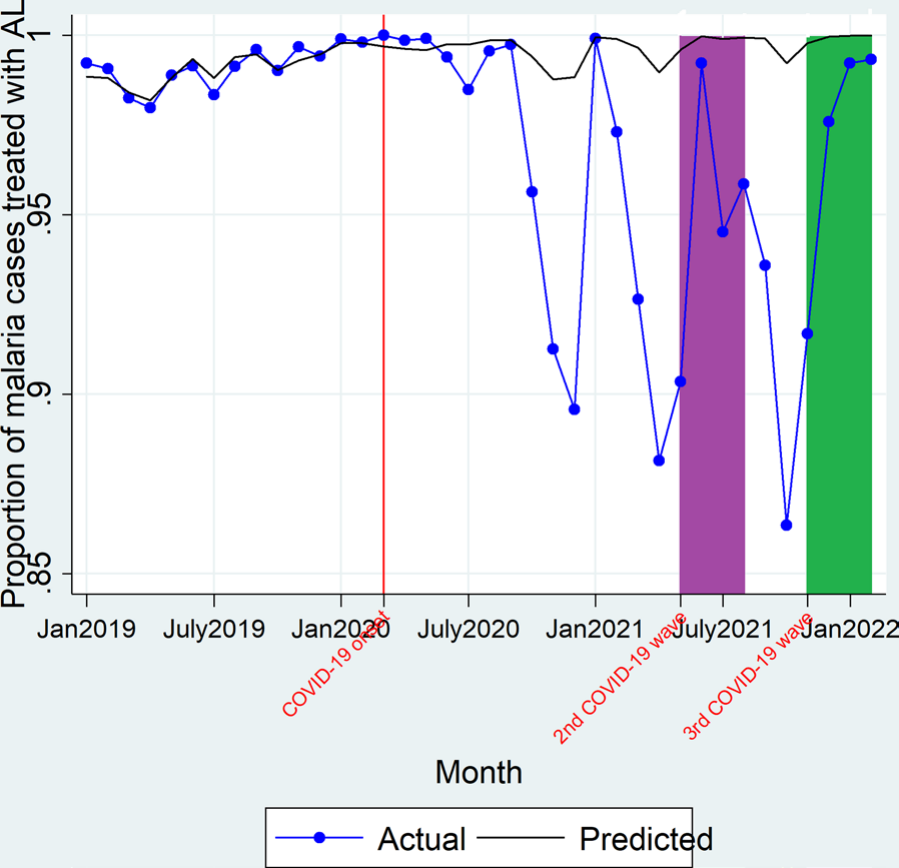
Actual and predicted proportion of confirmed malaria cases prescribed artemether lumefantrine. Red line denotes March 2020, the time when the country had its first COVID-19 cases and institution of restrictive measures on transport and lockdowns. Purple block depicts the duration when the country had the 2nd COVID-19 wave (May 2021–August 2021). Green block denotes the duration covered by the 3rd COVID-19 wave (November 2021–February 2022)

On onset of the 2nd COVID-19 wave, there was no immediate significant change in level (β_2_ = 3.33e−14, P > 0.05) of confirmed cases prescribed AL nor was there a significant change in trend (β_3_ = 7.66e−16, P > 0.05) of the proportion of malaria cases prescribed AL. There was no immediate significant change in level (β_2_ = 5.31e−14, P > 0.05) of the proportion of malaria cases prescribed AL on onset of the 3rd COVID-19 wave nor a significant change in trend of the proportion of malaria cases prescribed AL during the 3rd COVID-19 wave duration (β_3_ = 1.21e−14, P > 0.05) as shown in Fig. 5 and Table 2. Overall, there was a 5% decline (94.99% vs 99.85%; RPR = 0.95 [0.92–0.98]) in the proportion of malaria cases treated during the 2nd COVID-19 wave and a no significant difference between the observed versus predicted proportion of malaria cases prescribed AL during the 3rd COVID-19 wave (96.96% vs 99.93%; RPR = 0.97 [0.94–1.00]).

### Site specific impact

At Lumino HCIII, during the 2nd (90.57% vs 18.40%; RPR = 4.92 [− 6.85 to 16.69]), and 3rd (95.02% vs 0.24%; RPR = 397.28 [− 796.51 to 7811.09]) COVID-19 waves, there was no significant differences between the observed versus expected proportion of malaria cases prescribed AL. At Kasambya HCIII, there were no significant differences between the observed versus expected proportion of malaria cases treated for the 2nd (90.57% vs 18.40%; RPR = 4.92 [− 10.06 to 19.90]) and 3rd (95.57% vs 0.24%; RPR = 392.29 [− 5470.98 to 6215.56]) COVID-19 wave durations.

## Discussion

COVID-19 has been documented to negatively impact health care delivery and affect roll out of control interventions for several diseases including malaria. In this study, we assessed the impact of the 2nd and 3rd wave of COVID-19 on out-patient attendance, suspected malaria cases, test positivity rates and malaria case management.

### Summary of the results

During the 3rd COVID-19 wave, OPD visits were lower than expected, while no significant differences between the observed versus expected OPD visits were observed during the 2nd COVID-19 wave. However, at the two sites situated within moderate and low malaria transmission intensity settings, there was a significant decline in outpatient attendance during both the 2nd and 3rd COVID-19 waves. The observed proportions of suspected malaria cases were not significantly different from the expected during both the 2nd and 3rd COVID-19 waves, except at a site situated in a moderate malaria transmission setting where a decline was noted during the 3rd COVID-19 wave.

Test positivity rates remained consistent (no significant differences between the observed versus expected) overall, with significant increases during the 3rd COVID-19 wave at a site situated in a high malaria transmission setting and declines during the 2nd and 3rd COVID-19 waves at a moderate malaria setting situated site. The proportions of suspected malaria cases tested declined during the 3rd COVID-19 wave with no significant difference during the 2nd COVID-19 wave. The proportion of malaria cases prescribed AL proportions declined during the 2nd COVID-19 wave, with no significant difference during the 3rd COVID-19 wave, and no impact observed at sites situated in moderate and high malaria settings.

### Impact of the COVID-19 waves on outpatient malaria indicators, diagnostic and treatment practices

The reduction in OPD attendance (during both COVID-19 waves) at the sites situated in moderate and low malaria transmission settings could have resulted from either the instituted restrictions on travel as a measure for COVID-19 transmission reduction for the peri urban setting (low transmission situated site) and/or fear to contract COVID-19 on visiting the health facility [14, 15, 21]. Unfortunately, reductions in the number of patients seeking care at these public health facilities would mean that most people were staying at home even when they are getting ill and only presenting to the facilities when they have severe disease resulting into increases in complicated malaria cases and mortality (although this was not assessed as part of this study). It is also possible that due to COVID-19 stigma, people could have preferred to seek care from village health teams (VHTs).

A study conducted in Benin reported minimal effects of COVID-19 on health-seeking behaviour with some people reporting that they reduced how often they visited health facilities due to the COVID-19 pandemic and others saying that they did not change their health-seeking behaviour [22]. A retrospective analysis of routine surveillance data conducted in northern Ghana to determine the impact of COVID-19 on malaria reported a reduction in OPD visits during the 1st 6 months when COVID-19 restrictions were put in place and increases thereafter but still remained low relative to the previous years [23]. Another similar study conducted in Nigeria to determine the effect of COVID-19 on malaria intervention coverage also reported decline in care seeking practices across all age groups [24].

For the site located in a high malaria transmission setting were COVID-19 didn’t have effect on OPD attendance, it could be due to the fact that the site is located in a rural setting were people could still walk to the facility or use bicycles being a rural setting. The overall result (all sites combined) of decline in OPD attendance during the third COVID-19 wave would be due to the fact that the 3rd COVID-19 had more infectious cases, therefore people would have feared more to visit health facilities (in fear of contracting COVID-19) more in the 3rd COVID-19 wave relative to the other waves. However, overall (all sites combined), there wasn’t impact of COVID-19 on OPD attendance during the 2nd COVID-19 wave duration. This result is similar with that of the study conducted during the 1st COVID-19 wave in facilities located in the rural areas of Uganda, the study reported no impact on OPD attendance [16]. However, this disagrees with results from similar studies that reported decline in OPD attendance during COVID-19 [22, 23].

The overall (all sites combined), no effect of COVID-19 on the proportion of suspected malaria cases could be explained by change in the health-seeking behaviour of individuals within the communities were people feared to visit health facilities in fear of contracting COVID-19 or being classified as COVID-19 patients on presenting with fever [25, 26]. The suspected malaria cases/fever rates were expected to be high in all waves because both COVID-19 and malaria present with fever however, this was not the case, attributable to changes in health-seeking behaviour among the communities [26].

Also, during COVID-19, the prescription algorithm for COVID-19 was known in the communities, so even in cases where individuals got fever, they just bought the recommended drugs to handle COVID-19 or used lemons, oranges, ginger among others instead of visiting the health facilities. The fever cases could have been increasing within the communities but these were not being documented in the public health facilities because people were not visiting the health facilities as before out of the COVID-19 stigma. This result is similar to the result documented in the study conducted during the first wave in Uganda [16].

The overall (all sites combined) no effect of COVID-19 on the malaria test positivity rates at these facilities during the 2nd and 3rd COVID-19 waves could be explained by the fact that people with malaria fevers were not reporting to the health facilities out of fear of being taken to be COVID-19 suspects, therefore could remain home and self-medicate or could seek malaria care from the village health workers [25]. It could also have been due to the fact that people were entering their house early enough avoiding exposure to mosquitoes. People could have been seeking care from VHTs and benefiting from the Integrated Community Case Management (ICCM) which ensures malaria care especially in hard to reach areas [27]. It should also be noted that despite the delay in the third mass distribution campaign of mosquito nets that had to start in February 2020 but started later in June 2020 due to COVID-19 interruptions, the campaign was successful and ended in June 2021 ensuring continued protection from exposure to mosquito bites among communities explaining the no difference between the observed versus expected malaria test positivity rates [28].

This result is also similar to the results of a study done during the 1st COVID-19 wave in rural areas of Uganda which also reported no significant differences in the observed and expected TPR [16]. A similar study conducted in northern Ghana to determine the impact of COVID-19 on malaria reported that OPD and IPD malaria cases remained below during the pandemic relative to the previous years [23]. Another study conducted in in three malaria endemic districts of Rwanda to determine the effect of COVID-19 on malaria reported no change in the overall presentation rate of uncomplicated malaria and a reduction in the proportion of severe malaria [25].

However, at the site located in a high malaria transmission setting, the significant increment in the malaria test positivity rate during the 3rd COVID-19 wave could be associated with the fact that the duration of the 3rd COVID-19 wave coincides and/ or follows a rainy and malaria season in Uganda. However, it could also be due to a cumulative community buildup of malaria from the previous waves were people with suspected malaria could not visit facilities out of fear of being classified as COVID-19 suspects. A study conducted in Indochina an area that had a co-endemicity of COVID-19 and malaria showed an increment in malaria cases after removal of the lockdown and concluded that though lockdowns were effective in reducing COVID-19 transmissions, there removal was followed by increment in malaria cases [29].

When people do not access the services, malaria transmission in the communities increases. It is no wonder that after the third COVID-19 wave many malaria outbreaks have been noted in many parts of Uganda causing many malaria morbidities and mortalities [30]. This may be an impact of COVID-19 where the disease burden appeared to decrease as per health facility data yet it was increasing in the communities. The result of increment in the malaria test positivity rate during the 3rd COVID-19 wave in a high malaria transmission setting agrees with results of studies done in Zimbabwe and Central African Republic, which reported increment in malaria cases after onset of COVID-19.

It should however be noted that the study done in Zimbabwe which reported an excessive increment of malaria cases [31] did not control for environmental factors, specifically rainfall and temperature, that are known covariates of malaria in that country. The reported increase in malaria morbidity and mortality also coincided with the malaria peak season in that country. The study done in Central African Republic reported increment in the prevalence of asymptomatic malaria from August to September 2021 compared to a similar study done before COVID-19 [32].

The result on overall (all sites combined) decline in the proportion of suspected malaria cases tested (using both RDTs and microscopy) reported in this study agrees with a result of a similar study conducted in Senegal that aimed to determine the impact of COVID-19 on biological diagnosis of malaria which reported a decline in the malaria tests done (both RDTs and microscopy) in 2020 COVID-19 year relative to the prior years [33]. Another study conducted in Mozambique to determine the impact of COVID-19 on malaria surveillance with a specific focus on diagnosis and treatment reported a decline in the number of people tested for malaria in the health facilities and an increase in the number tested for malaria in the communities [34].

This result on overall (all sites combined) decline in the proportion of suspected malaria cases tested (using both RDTs and microscopy) during the 3rd COVID-19 wave duration could have been due to shortage on malaria rapid diagnostic test kits since most biomedical firms shifted focus to producing COVID-19 rapid diagnostic test kits [35]. The decline could also be due to absentia of health workers at the facilities due to either COVID-19 stigma or lack of transport however much the government waived their movement despite the travel restrictions. It could also be that people were seeking care from the VHTs. A similar study conducted in 3 malaria endemic districts in Rwanda to determine the impact of COVID-19 on malaria services reported a decline in malaria testing at the health facilities and an increment in malaria testing at community level which they attributed to COVID-19 mitigation measures such as travel restrictions but also highlighted people’s fear to contract COVID-19 on visiting the health facilities [25].

During the 2nd COVID-19 wave duration, there was no significant difference (all sites combined) between the observed versus expected proportion of suspected malaria cases tested (which is also true for the site located in the high malaria transmission setting throughout both the 2nd and 3rd COVID-19 waves) which could be attributed to campaigns such as “Why survive COVID-19 and die of malaria?” which were put in place ensuring the malaria testing of all fever cases at the health centres [36]. Health workers were re-trained, redistributed and reassigned to ensure adherence to malaria testing of all fever cases in an attempt to prevent the disease from re-emerging due to focus shift to COVID-19.

The significant decline in the proportion of malaria cases treated with artemether-lumefantrine (first-line anti-malarial treatment of uncomplicated malaria in Uganda) during the 2nd wave duration would have been due to a run out on supply of AL due to the shift of the resources to fight COVID-19. The 1st COVID-19 study also reported similar findings [16]. Evidence has shown that access to anti-malarials was disrupted in sub-Saharan Africa during COVID-19 [37].

### Strength of the study

This study had strength when compared to most studies done in malaria endemic countries to assess the impact of COVID-19 on malaria in that seasonality and the most important covariates of malaria predominantly rainfall and temperature were adjusted for when predicting the study outcomes had the second and third COVID-19 waves not occurred. If not controlled for, these could confound the study results. Interrupted time series analysis used to assess the impact of COVID-19 on malaria in this study has beauty of taking the pre-COVID-19 malaria trends into consideration and also producing counterfactual trends if at all COVID-19 had not occurred. Most of the studies done to assess the impact of COVID-19 were done in the first wave when COVID-19 cases were still few but this study covered both the second and third COVID-19 waves were COVID-19 cases were at peak and more infectious. The sample size used was 18 times the estimated that the study had a final power of more than 99%.

### Limitations

However, there were still limitations, only three study sites were purposively selected to represent low, moderate and high malaria transmission settings of Uganda, but more sites would have been chosen to represent each malaria transmission setting. On calculating outcome estimates had COVID-19 not occurred, since only rainfall, temperature and calendar month effects were adjusted for leaving out other environmental covariates of malaria including humidity, vegetation index among others. Disparities in IRS status and LLIN distribution status across the study sites was not taken into account. There remains a question on the completeness of the data even in the case where surveillance data was used in this study. Absence of health workers and data officers during COVID-19 due to either COVID-19 stigma and/or difficulty in movement could also have impacted the quality of the data. Single group ITSA also has a limitation of lack of controls.

## Conclusions

The 3rd COVID-19 wave was associated with a significant reduction in outpatient department attendance. Subgroup analysis however showed consistent negative impact across both the 2nd and 3rd COVID-19 waves at a low and moderate malaria transmission situated sites. While there were no significant changes in the proportion of suspected malaria cases and test-positivity rates overall, subgroup analysis showed varying effects, including a significant increase in test-positivity rates during the 3rd COVID-19 wave at a high malaria transmission situated site and declines in both test-positivity rates and proportion of suspected malaria cases during the 2nd and 3rd COVID-19 waves at a moderate malaria transmission situated site. Additionally, there were notable impacts on malaria diagnostic practices during the 3rd COVID-19 wave unlike the 2nd COVID-19 wave and impacts on the antimalarial (artemether lumefantrine) prescription practices during the 2nd COVID-19 wave unlike the 3rd COVID-19 wave. This means there is need to bring the malaria services near to the communities during out-breaks like COVID-19 so that care is not disrupted. If this intervention is not done, there would be more severe disease and even increased malaria mortalities in the communities.

### Recommendations

It is recommended that in case of any other outbreak, all efforts should be made to ensure continuous delivery of malaria services. This can be done through strengthening and extending the integrated case community management for malaria in all districts such that in circumstances where people fear to visit health facilities in fear of contracting an emergent disease such as COVID-19, they can still access malaria care at community level. The MoH should sensitize the public about the changes in health-seeking behaviour that happened during the lockdowns to encourage people seek medical care again. There is need for a more extensive study with data from more health facilities within each malaria transmission setting and covering the entire COVID-19 duration to either refute or to agree with the results of this study. Other studies should be also be conducted to determine the impact of COVID-19 on malaria at community level. Studies should also look at the burden of severe malaria that happened during COVID-19 comparing them with the pre- COVID-19 period.

## Data Availability

The datasets used/analyzed in the study are available on request from the corresponding author.

## Abbreviations

AL: Artemether Lumefantrine
HC: Health Centre
HIV: Human Immunodeficiency virus
IPTp: Intermittent Preventive Treatment for Pregnancy
IRS: Indoor residual spraying
ITN: Insecticide treated net
ITSA: Interrupted time series analysis
LLINS: Long lasting insecticidal nets
MoH: Ministry of Health
OLS: Ordinary least squares
OPD: Outpatient department
RDT: Rapid diagnostic test
TB: Tuberculosis
TPR: Test positivity rate
UMIS: Uganda Malaria Indicator Survey
WHO: World Health Organization
